# Neural and Mental Hierarchies

**DOI:** 10.3389/fpsyg.2012.00516

**Published:** 2012-11-26

**Authors:** Gerald Wiest

**Affiliations:** ^1^Department of Neurology, Medical University ViennaVienna, Austria; ^2^Vienna Psychoanalytical SocietyVienna, Austria

**Keywords:** hierarchies, evolution, mind, brain, self, behavior, mental, psychoanalysis

## Abstract

The history of the sciences of the human brain and mind has been characterized from the beginning by two parallel traditions. The prevailing theory that still influences the way current neuroimaging techniques interpret brain function, can be traced back to classical localizational theories, which in turn go back to early phrenological theories. The other approach has its origins in the hierarchical neurological theories of Hughlings-Jackson, which have been influenced by the philosophical conceptions of Herbert Spencer. Another hallmark of the hierarchical tradition, which is also inherent to psychoanalytic metapsychology, is its deeply evolutionary perspective by taking both ontogenetic and phylogenetic trajectories into consideration. This article provides an outline on hierarchical concepts in brain and mind sciences, which contrast with current cognitivistic and non-hierarchical theories in the neurosciences.

## The Philosophical and Biological Foundations of a Theory

According to modern biology, the development of hierarchies distinguishes the organic from the anorganic world (Mayr, 1997). Herbert Spencer (1820–1903) was the first who – influenced by Lamarckism – provided a coherent theory on the evolution, structure, and function of the nervous system. In his “Principles of Psychology” (Spencer, 1855), he postulated that the human mind can only be fully understood by considering its phylogenetic development. In his view, the phylogeny of consciousness illustrates a general principle of evolution, namely the development from a simple, undifferentiated homogeneity to a complex, differentiated heterogeneity. This conception implies that the human mind had evolved in the same way from a simple automatic response in lower animals to higher cognitive processes in man.

**Figure 1:**
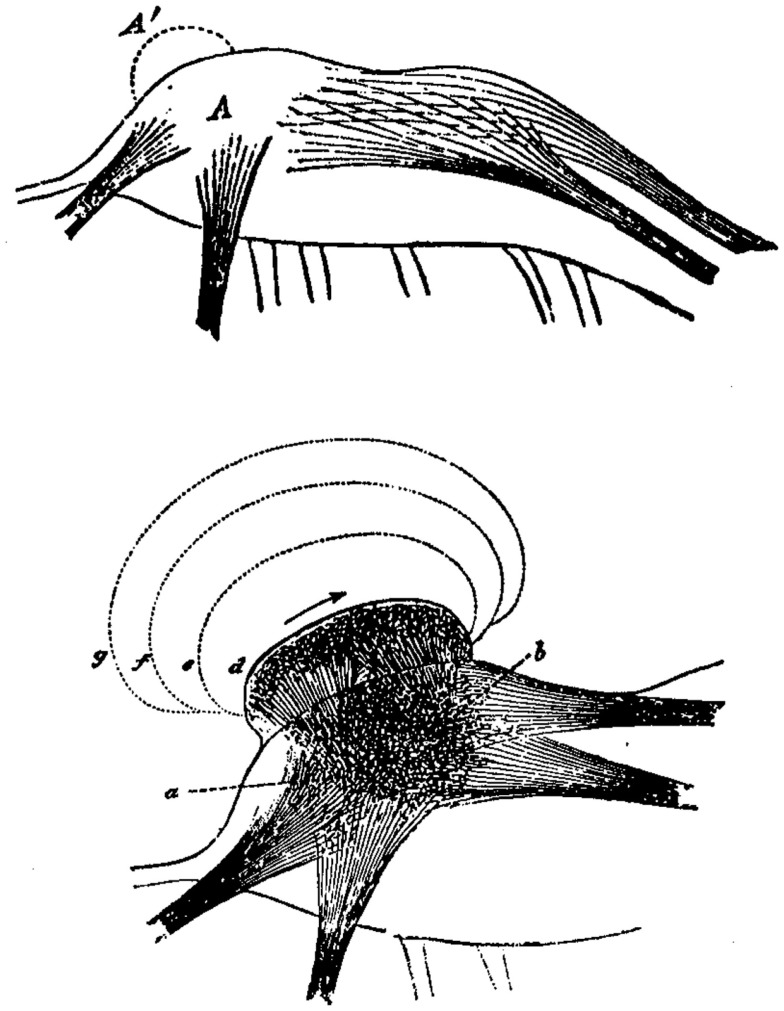
**Herbert Spencer’s concept of the evolutionary change in the nervous system by means of superimpositions (A′) of neural layers exemplified in a neural ganglion (A)**. Consequently, neural excitation does not proceed anymore from *a* to *b* but is rather following new neural formations (*d, e, f, g*). Image from Spencer H (The Principles of Psychology, 1896, Appleton Press).

Spencer envisaged the evolutionary change of neural structures toward higher complexity as a process of stratification or layering of neural formations (Figure 1). Thus, each neural formation of the nervous system not only represents impressions and experiences of the individual’s past, but also those of its ancestors. From a neurological perspective, this would mean that a lesion at a higher cerebral level unveils neural and mental functions from an earlier evolutionary stage.

The British neurologist John Hughlings Jackson (1834–1911), one of the founding fathers of clinical neurology, was intrigued by the correlations between Spencer’s proposed evolutionary principles of neural functioning and his clinical observations in patients with focal brain lesions. In contrast to Spencer’s interest in evolutionary aspects of brain function, Jackson was more occupied with the reverse effect of evolution, which he coined “dissolution.”

In Jackson’s view, neurological symptoms such as aphasia, hemiparesis, or epileptic seizures, represent a dissolution, i.e., a reversal of the evolution of the nervous system, caused by a cerebral lesion. Jackson found that evolutionary higher cerebral centers inhibit the lower ones and lesions at these higher centers are accompanied by the production of “negative” symptoms (e.g., a palsy due to the absence of function) and of “positive” symptoms (e.g., pyramidal signs), caused by a functional release of the lower centers. Neurological or psychiatric symptoms can in this regard provide a look into the phylogeny of neural function. In his major work entitled “Evolution and Dissolution of the Nervous System” (Taylor, 1931/1932) Jackson outlined his theory of brain function, which still belongs to the foundations of neurology. The validity of a hierarchical organization of the nervous system has subsequently been confirmed by modern neurology and neuroscience for a variety of neural systems (Kennard, 1989; Swash, 1989; Vallbo, 1989; Miller and Cohen, 2001; Greene et al., 2004). The Jacksonian concept contrasts with non-hierarchical models of brain function, such as the theories by Hebb (Brown and Milner, 2003) or Lashley (1958). These models propose that brain function, in particular cortical processing, is based on the distributed processing of cell assemblies, i.e., of neural networks. According to lesion studies, Lashley proposed for example, that memories are not localized but widely distributed across the cortex, which has not been confirmed in subsequent studies. Classical empirical studies as well as recent imaging studies – on the other hand – provide convincing evidence that the rostro-caudal axis of the frontal lobe may indeed be hierarchically organized (Goldstein and Scherer, 1941; Luria, 1966; Mesulam, 2002; Petrides, 2005; Badre and D’Esposito, 2009). However, the Jacksonian concept not only paved the way to the establishment of neurology as a scientific discipline, it also had a profound impact on Sigmund Freud and the development of psychoanalytic metapsychology.

## Dissolutions in the Nervous System

Human brains and that of higher mammals share many structural and functional commonalities. However, the comparison of common behavioral patterns, in particular that of inherited or instinctive traits, is hampered by the fact that these phylogenetically old features can not be examined or assessed by conventional experimental designs or psychological methods. Instead, one has to rely on comparative morphology and on the methods of comparative ethology for the evaluation of homolog instinctual behavior. Pure instinctual behavior in humans can only be observed during early infancy. These instinctual motor phenomena, also referred to as “primitive reflexes” in clinical neurology, usually disappear as the child progresses through the stages of movement development. This means that the vanishing of primitive reflexes is closely related with the maturation of the nervous system, in particular the frontal cortex, which obviously exerts an inhibitory effect on these instinctual reflexes. The gradual replacement of inherited and automatic movements by intentional and goal-directed movements reminds one of Haeckel’s proposal, that in human development ontogeny recapitulates phylogeny, at least to some extent (Haeckel, 1866, 1874). This developmental peculiarity confirms, on the other hand, that motor development and motor organization obeys hierarchical principles, i.e., that higher or mature motor systems control or inhibit lower and automatic ones.

Another possibility to observe primitive reflexes in humans, is to look at patients with focal brain lesions. According to the Jacksonian principle of dissolution, lesions at specific brain sites may release these reflexes. As mostly frontal brain lesions give rise to this releasing phenomenon, primitive reflexes are also referred to as “frontal release signs.” Depending on the degree of brain damage the primitive reflexes may be present automatically or they can only be elicited by stimulation, i.e., that they manifest themselves reflectively.

Oral or manual grasping reflexes are one of the best known primitive reflexes (Pilleri and Poeck, 1964). They can be observed both in newborn humans and in young primates, as well as in patients with diffuse brain damage, such as extended frontal brain injury or neurodegenerative diseases (e.g., Alzheimer’s disease). These reflexes provide an evolutionary advantage in early infancy for the otherwise helpless child, as the oral grasping reflex enables the newborn to find the breast and the nipples, the manual grasping reflex likewise enables the infant or the newborn primate to cling to its mother’s body or fur, respectively.

Disinhibitory phenomena or release mechanisms are not restricted to the reflex level, they can also be observed in the motor/action control system, as well as in the sexual and affective domain. Utilization behavior, for example, is a clinical sign in which the visuo-tactile presentation of objects compels patients to grasp and use them, despite not being instructed to do so (Lhermitte, 1983, 1986a; Lhermitte et al., 1986b). This behavior persists even if the examiner asks them to stop and is usually associated with frontal lobe lesions. The symptom is considered to result from an impairment of the capacity to inhibit actions triggered by the perception of objects (Besnard et al., 2010). Shallice et al. (1989) proposed in this regard a hierarchical model of action control. The lowest level of the model includes “*action schemas*,” defined as abstract representations of well-learned action sequences that are selected once the activation level exceeds a “threshold” that depends on environmental stimulation. At the highest level of the model, the “ *supervisory attentional system*” (SAS) – thought to be located in the frontal lobes – has a monitoring function that includes planning, decision-making, or suppressing a dominant response.

Brain lesions associated with disinhibited sexual or aggressive behavior, on the other hand, are usually located in the medial temporal lobes (Bingley, 1958). Pathological laughter and crying represent disorders of emotional expression and are characterized by uncontrollable episodes of laughter or crying. Pathophysiologically, it is assumed that these symptoms are caused by a damage of pathways descending from cortical motor areas to a presumed center for laughter and crying in the brainstem. The symptomatology of pathological laughter and crying can thus also be understood as disinhibition or releasing phenomenon of the emotional motor system (Poeck, 1985).

## Three Brains in One

Classical ethology defined instincts as hierarchically organized mechanisms of the nervous system, which are triggered by specific external stimuli (Tinbergen, 1951). A more detailed hierarchical model of instinctual behavior has been proposed by the ethologist Baerends (1956) by differentiating between “higher” and “lower” instincts and “fixed action patterns.” Baerends also assumed that hierarchically lower centers are being controlled by multiple higher centers.

**Figure 2:**
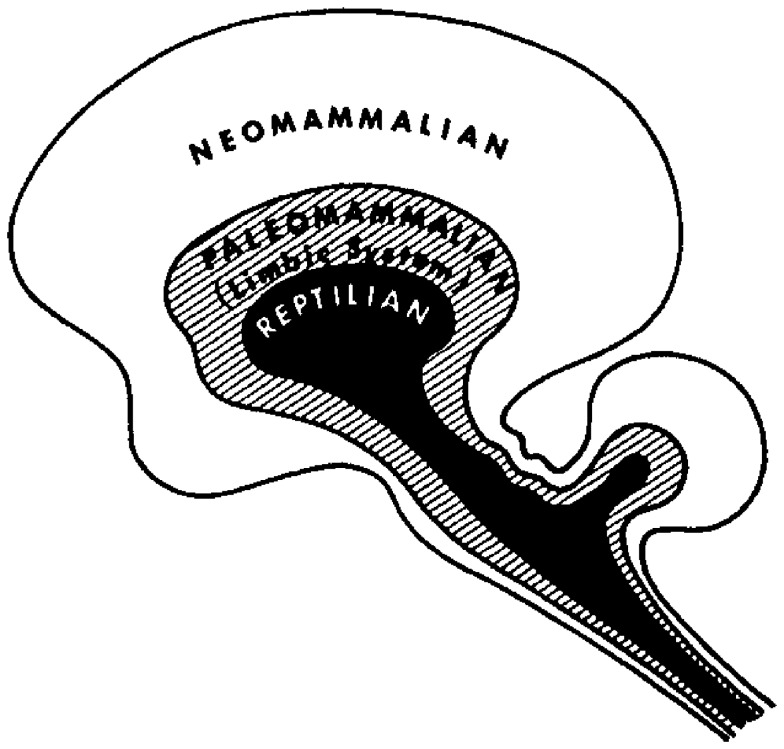
**The Triune Brain Model by MacLean (1990)**. Image courtesy of Springer Verlag.

A more sophisticated hierarchical theory of brain function, which incorporates both neural morphology and evolutionary aspects of behavior, has been put forward by the neuroscientist Paul MacLean. His theory, which is also known as the “triune brain theory” (MacLean, 1990), is based on the assumption that the human brain actually integrates three different brains, i.e., that each “brain” represents a specific hierarchic or evolutionary level, ranging from an ancient “reptilian” brain to a “paleomammalian” brain and a “neomammalian” brain (Figure 2). However, it would be too simplistic to conceive these different “brains” as purely superimposed neural layers. Instead, the different brains seem to cooperate like “three interconnected biological computers,” each of them having its own feeling of subjectivity and its own perception of time, space, and memories.

The reptilian brain, or R-complex, is the oldest part in the triune brain model and represents the brain of reptiles (therapsids) that preceded mammals (190 million years ago). Homologous formations of the R-complex can be found in some structures of the human basal ganglia, the principle structure of the extrapyramidal motor system. Naturally, this oldest part of the forebrain is involved in instinctual, ritualistic, and routine behavior. Furthermore, it is essential for the controlling of fighting and mating behavior. As the reptilian brain is lacking the ability to communicate (reptiles are for example unable to communicate with their offspring) Paul MacLean denominated the mental functioning of the R-complex as “protomentation.” The method of comparative ethology provides a way to delineate R-complex-associated homologous behavior in different species. The R-complex controlled behavior of territoriality, for instance, can be observed in reptiles, lower and higher mammals, as well as in humans. The same applies for so-called behavioral or daily routines that can be found both in reptiles and mammals, as well as in humans; the term “habits” would be more suitable in the last case, though (Graybiel, 2008).

The paleomammalian brain is in contrast to the reptilian brain a more recent achievement of brain evolution. The neural substrate of the paleomammalian brain correlates roughly with what is commonly referred to as the limbic system (Nieuwenhuys et al., 2007). The limbic system may be called the common denominator of all mammals, as it is the essential area in the brain for bonding, attachment, and emotions. These new evolutionary features of the mammalian brain are vital for the care of their offspring and thus for the survival of the species. Experimental studies in animals have shown that the limbic structures monitor both cues from the external world and internal signals from inside the body and orchestrate their interoperation (Hebben et al., 1985). Experimental phenomena in patients with temporal lobe epilepsy and electrical stimulation studies of the human limbic system have confirmed that this area of the brain is critical for a variety of functions, such as the processing of emotions, the evaluation of other’s intentions (Adolphs et al., 1994, 1999), or the consolidation of affective and autobiographic memories (McGaugh, 2004; Buchanan et al., 2005; Wiest et al., 2006). It seems as if particularly the capacity to encode emotionally salient memories or of ongoing experiences contributes significantly to the specific human perception of individuality (MacLean, 1969). MacLean introduced the term “emotomentation” to describe the mental activity and characteristics of the paleomammalian brain. The main hallmark of the limbic system is that it regulates behavior according to emotional cues (Morgane et al., 2005).

Brain evolution culminated finally in the emergence of the neocortex, which – unlike the limbic system – underwent massive expansion in higher mammals. Accordingly, MacLean coined the term “neomammalian brain.” The latin term “pallium” underlines its anatomical peculiarity, namely that it encases both the reptilian and the paleomammalian brain like a coat. Due to its high connectivity the neocortex is specialized in the integration of multisensory information and it is the seat of language, abstraction, and planning. These qualities ultimately enable higher mammals to solve problems and humans to apply symbolic reasoning. These high-level processes are also critical for the development of unique human social capacities such as altruism, cooperation, or empathy.

MacLean’s triune brain theory not only enriched the brain sciences by introducing the new field of evolutionary neuroethology, it may also help bridging the gap between neuroscience and dynamic and evolutionary sciences of the mind, such as evolutionary psychiatry, psychoanalysis, or dynamic neuropsychological theories.

In 1977 Paul MacLean already emphasized that the lack of similar chemistry and anatomy of the three evolutionary formations may give rise to communicative conflicts between these systems (MacLean, 1977). The new discipline of Evolutionary Psychiatry applies the triune brain model for the explanation of a variety of psychiatric disorders. In this view, psychiatric symptoms are manifestations of ancient adaptive strategies which are no longer appropriate but which can be best understood and treated in an evolutionary and developmental context (Stevens and Price, 2000). Symptoms like depression or anxiety disorders may in this context be understood as maladaptive expressions of adaptive communicational states exhibited normally in many species. Thus, depressive mood in humans may resemble submissive behavior to conspecifics as found in many animals. In group-living animals, submissive behavior finally serves to sustain the hierarchical structure of the group, and is thus an adaptive behavioral trait (Price, 1967, 2002; Price et al., 1994).

It should not be unmentioned that the triune brain theory has been criticized for being too simplistic or for being not compatible with current evolutionary theories (Butler and Hodos, 2005). From a psychoanalytic perspective MacLean has been criticized for using Jacksonian concepts to separate reason from emotion and to suggest that rational behavior is “better” than emotional behavior (Modell, 2000). However, MacLean’s work, which – after all – is based on experimental data, paved the way to affective neuroscience, a meanwhile established field in the neurosciences. In his book “Affective Neuroscience” Jaak Panksepp refers to MacLeans triune brain theory: *“This three-layered conceptualization helps us grasp the overall function of higher brain areas better than any other scheme yet devised. Of course, exceptions can be found to all generalizations, and it must be kept in mind that the brain is a massively interconnected organ whose every part can find an access pathway to any other part. Even though many specialists have criticized the overall accuracy of the image of a “triune brain,” the conceptualization provides a useful overview of mammalian brain organization above the lower brain stem”* (Panksepp, 1998).

Recently, Jaak Panksepp proposed a neuroevolutionary model of the emotional system, which rests on the theories of Jackson and MacLean. According to findings from electrical brain stimulation Panksepp proposes that instinctual emotional behaviors and feelings emanate from homologous brain functions in all mammals, which are regulated by higher brain regions. Such findings suggest nested-hierarchies of affective processing, with primal emotional functions being foundational for secondary process learning and memory mechanisms, which interface with tertiary-process cognitive-thoughtful functions (Panksepp, 2011). An extensive reappraisal of the validity of MacLeans theories has been conducted by Gerald Cory in his book “The Evolutionary Neuroethology of Paul MacLean: Convergences and Frontiers” (Cory and Gardner, 2002).

## The Hierarchic Mental Apparatus

The main focus of psychoanalysis is the investigation of the individual subject and its relations to the external and internal world. According to this comprehensive approach, psychoanalysis still represents for many scientists the most coherent and intellectually satisfying view of the mind (Kandel, 2006). The primary interest of psychoanalysis in the subjective experience of the self correlates to some extent with the research areas of evolutionary neuroethology. In the introduction of his book “The triune brain” (MacLean, 1990) MacLean noted, that there exists no branch of (empirical) science that deals specifically with an explanation of the subjective self and its relation to the internal and external environment. He proposed that a science dealing with this topic could be referred to as “epistemics.” It is probably no coincidence in this regard, that both evolutionary neuroethology and psychoanalysis are based on the evolutionary and hierarchical conceptions of Spencer and Jackson. In his book on aphasia (Freud, 1891) Freud adopted as a guiding principle Jackson’s doctrine that all phenomena observed in aphatic patients represent instances of functional retrogression of a highly organized apparatus and that the symptoms of aphasia correspond with earlier states of its functional development. Hierarchical principles can be found in a variety of psychoanalytic concepts: (1) in the metapsychological models of the mental apparatus, (2) in the two principles of mental functioning, i.e., the primary and the secondary process, (3) in the concept of regression. Regarding the psychoanalytic concepts of the development of the mental apparatus there are some parallels with the Jacksonian model of neural maturation. During brain development early modes of behavior come under the controlling influence of higher centers. This process also involves progression from automatically organized behavior to voluntary control.

In his topographical model of the mental apparatus Freud proposed a system of three separated compartments (system unconscious, system preconscious, and system conscious). Similar to the triune brain theory, the compartments are not operating in complete isolation, rather they have to be imagined as a continuum. A hierarchical organization of the mind is also inherent to primary and secondary process mentation. MacLean himself pointed to the analogies between the averbal communicative features (which he called “prosmatic” communication) of the reptilian and paleomammalian brain and the Freudian primary process (MacLean, 1990). Human mental development progresses from mostly primary to secondary process functioning, i.e., that infancy and childhood are characterized by the prevalence of drive- or instinctual-based wishes, while the capacity to contact and to deal with reality improves with age. Freud developed the topographical model of the mind as a reference frame for the description of mental functions. Similar to Spencer he conceptualized a model that is based on the degree of accessibility of specific mental activities to consciousness. According to Spencer, during phylogenetic development archaic structures of consciousness are increasingly being inverted into reflex centers, making these lower qualities of consciousness inaccessible to higher centers: *“Beyond the limits of the coherent aggregate of activities*…*constituting consciousness, there exist other activities of the same intrinsic nature, which being cut off are rendered foreign to it”* (Spencer, 1855).

A hierarchical organization is also inherent to Freud’s structural model of the mental apparatus (Freud, 1923, 1926). While the topographical model’s domains are conscious and unconscious processes, the structural model’s focus lies on the psychological conflicts between the “structural” components (id, ego, and super-ego) of the mental apparatus. Several authors have tried to find correlations between the hierarchical principles in brain organization and psychoanalytic metapsychology. David Rapaport’s influential theory of thinking is, for example, based on hierarchical concepts:

*“We have assumed that the organization of cathectic energies is a hierarchy in which the forces of the basic energy distribution are controlled by a superimposed one arising from it, which in turn gives rise to another set of forces which are then similarly controlled, and so on; we assume that thought organization also follows this hierarchic layering”* (Rapaport, 1951). Psychoanalysts have also suggested that developmental psychology in general, as well as the phylogenetic succession of involuntary to voluntary mental processes can be thought of as hierarchical (Wilson and Gedo, 1992; Modell, 2000). Tarpy (1977) pointed to the similarities between the limbic system and the id, as well as between the cortex and the ego. In a more comprehensive approach, Fishbein (1976) and Ploog (2003) were one of the first to combine the findings of evolutionary neuroethology with the psychoanalytic concepts of the mental apparatus and with psychiatric disorders. More recent neuroimaging studies provide convincing data that the Freudian concepts of the mental apparatus have neurobiological substrates. If the brain is conceived as a hierarchical inference machine (Helmholtz machine), then large-scale intrinsic networks occupy supraordinate levels of hierarchical brain systems that try to optimize their representation of the sensorium. In this view, the Freudian concepts of primary and secondary processes would correlate with self-organized activity in hierarchical cortical systems. Likewise, Freud’s concept of the ego would be consistent with the functions of the so-called default-mode and its reciprocal exchanges with subordinate brain systems (Carhart-Harris and Friston, 2010). From a Jacksonian perspective, these intrinsic networks correspond to the high-levels of an inferential hierarchy, which function to suppress the free-energy of lower levels.

The term regression generally denotes a return to a lower level. In psychoanalysis, regression refers to a return to an earlier stage of development, which implies a hierarchical structure. Freud’s structural model is conceived as a system in which the ego and super-ego controls or inhibits the lower components of the apparatus, i.e., the id. Under certain conditions, the controlling influence becomes impaired and previously inhibited infantile behavior patterns re-emerge. This regressive process represents a reversal of the developmental process, or a dissolution in the sense of Spencer and Jackson (Goldstein, 1995). Irrespective of the various forms of regression, like topic, temporal, or formal regression, the process itself is one of the core concepts of psychoanalysis in terms of the etiology and symptomatology of neurotic disorders (Jackson, 1969). Gedo and Goldberg (1973) proposed a model of psychic development which illustrates the maturational level of the mental apparatus over time and thus allows a precise assessment of an individual’s level of regression.

## Layers of the Mind

The scientific discipline of neuropsychology evolved primarily for the purpose of evaluating the effects of brain damage on psychological functioning. Studies in patients with focal brain lesions at the end of the nineteenth century paved the way to classical localizational theories in neuropsychology. Alternative approaches to the understanding of psychological symptoms are based on theories that incorporate evolutionary and maturational trajectories. Several neuropsychological symptoms with particular phenomenology cannot be understood in a traditional way as a pure deficit syndrome. The application of dynamic/hierarchical concepts in these cases provides a coherent explanation of both negative and positive components of the disorder.

So-called neuropathologies of the self represent a particular group of psychological deficits that cause a profound and specific alteration in a patient’s identity (Feinberg, 2001). Furthermore, these patients exhibit a disorder of the relationship between the self and the world, which may affect the bodily self, the relational self, or the narrative self. Interestingly, all these delusional states do have one thing in common: the symptoms are restricted to something of personal significance to the individual (Feinberg and Roane, 1997). The clinical spectrum of these neuropathologies of the self range from delusions and confabulations (Fotopoulou, 2010; Kopelman, 2010) to misidentifications or paramnesias.

**Figure 3:**
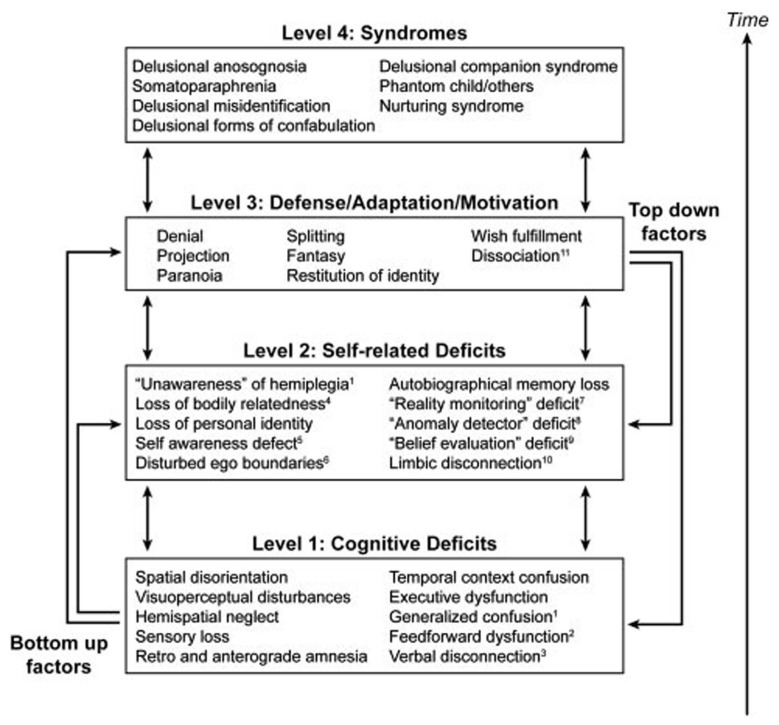
**A hierarchical four-tiered model of representative factors contributing to the neuropathologies of the self**. Specific cognitive deficits may only be relevant to certain conditions, while self-related deficits and positive features may be applied to all syndromes (Feinberg, 2011). Image courtesy of Elsevier.

In his “disequilibrium theory” Feinberg (2009) proposes that neuropathologies of the self are neurologically induced alterations of the self boundaries that results in a regression to a developmentally earlier, hierarchically lower, or more primitive stage of psychological functioning that causes a recurrence of the patterns of thought and psychological defense typical of these earlier periods. The primitive defense mechanisms (in a psychoanalytic sense) associated with these disorders are denial, projection, splitting, fantasy, and paranoia. The theory implies, that these functions were dormant in the normal adult brain but are now activated by the neurological lesion. A hierarchical model to account for the characteristics of the neuropathologies of the self has been put forward by Feinberg (2011) with three levels of interacting factors and the final syndrome in question on the top tier (Figure 3). The peculiarity of Feinberg’s approach is the application of genuine psychoanalytic terminology and concepts in neuropsychological disorders, thus combining the ideas of Spencer, Jackson, and Freud (Solms, 2010). However, it is not Feinberg’s intention to apply his disequilibrium theory and the concept of regression to the whole of neuropsychology, but rather specifically to the neuropathologies of the self, which may be classified as “hybrid” of a neurological and psychiatric disorder. As a consequence, Feinberg conceptualizes the classical neuropsychological syndromes, such as aphasia, apraxia, or anosognosia, as neutral with reference to the patient’s personal relatedness to self and world. The latter view, however, is not in line with the neuropsychoanalytic findings by Kaplan-Solms and Solms (2002). Feinberg’s model has been criticized by some authors for being too structurally oriented and less dynamic. In contrast, Salas and Turnbull (2010) proposed a more psychodynamic approach, suggesting that the recrudescence of primitive defense mechanisms in the neuropathologies of the self may be caused by a failure in the capacity to regulate the intensity of arousal and negative emotional states. Thus, from this perspective it is not that primitive defenses “take over” due to damage to the right hemisphere, but that this damage impairs the arousal regulation capacity, which forms one component of mature defenses.

Lesions associated with neuropathologies of the self are usually located exclusively in the right frontal lobe, suggesting that this area plays a critical role in the establishment of ego boundaries and to mediate the relationship between self and world. In the rare delusional syndrome somatoparaphrenia the relationship between the self and the body is disturbed, i.e., these patients claim that their left paralyzed arm belongs to someone else. In a recent study, Fotopoulou et al. (2011) have demonstrated, that this limb-disownership can be altered by using self-observation in a mirror, i.e., by switching from a first- to a third-person perspective. These findings imply that somatoparaphrenia represents a neurologically induced dissociation between the felt and seen body. From a hierarchical and Jacksonian point of view, the symptoms of somatoparaphrenia can be understood as an impairment in the integration and higher order re-representation of bodily signals. Thus, lesions in these higher centers unfold older and disintegrated or dissociated representations of body ownership, just like an infant that is not yet able to integrate its felt body with its disembodied image in a mirror.

Microgenetic theory represents another hierarchical concept of neural functioning that has been developed in neurological patients with specific brain lesions. The basic assumption in microgenetic theory is that all mental activities, i.e., actions, perceptions, thoughts, affects, memories, and even consciousness, are the result of an unfolding process. This process occurs within a fraction of a second always in a bottom-up direction, along the evolutionary trajectories of the brain. In this view, mental representations and actions unfold from depth to surface, i.e., that phylogenetic and ontogenetic growth patterns are retraced in microgeny (Brown, 1998). The Jacksonian theory proceeds from the assumption that developmentally early stages elaborate automatic processes, whereas higher levels elaborate volitional performances. Thus, in this model function is not transferred but re-represented again at higher levels in a different form. Microgenetic theory, on the other hand, proposes that preliminary stages are not released or disinhibited from above, but exhibit a form of cognition consistent with a certain level of derivation (Brown, 1988). It has been suggested that so-called event-related potentials, which can be recorded from the cortex during specific mental processes or cognitive tasks, may represent the neural correlate of the microgenetic process. In this view, neurological symptoms can be conceived as disruptions of a microgenetic process, i.e., that the unfolding of the process is stopped at a certain level and only parts of it are reaching the cortical level (Brown, 1988).

## Conclusion

Hierarchical models of the brain and the mind can be found in a variety of scientific disciplines. Based on the foundational theories of both Spencer and Jackson the spectrum of hierarchical concepts ranges from such diverse fields as neurology, neuropsychology, and ethology to psychoanalysis and microgenetic theories. A hallmark of the theories outlined in this paper is that hierarchical organizational principles can be equally applied to aspects of brain and mind functions, which contrasts with the prevailing reductionism in the neurosciences. The paper supports the idea that the brain can not be reduced to a pure information processing device, but that all neural and mental functions can only fully be understood if their evolutionary trajectories have also been taken into consideration. Thus, a specific feature of the hierarchical concepts outlined in this paper is the integration of ontogenetic and phylogenetic aspects of brain and mind functions, which emphasizes the deeply evolutionary approach in these theories. The evolutionary layered nature of brain organization also implies that one has to overcome the traditional Cartesian dualities of mind and brain in the neurosciences. The discipline of Neuropsychoanalysis (Panksepp and Solms, 2012) and the Affective Neuroscience of Panksepp (1998), for example, propose a monistic approach to the understanding of the mind. Accordingly, the brain is not conceptualized as a sensorimotor machine but as a unified experience-generating organ. In this regard, the term BrainMind or MindBrain, has recently been coined in order to overcome the Cartesian dilemma of the brain-mind dualism (Panksepp, 2011). Based on this tradition, the current paper is an attempt to demonstrate that hierarchical concepts from different scientific disciplines facilitate an integrative view of the mind/brain relationship and that these theories may represent a common denominator for the sciences that deal with the nature of brain, mind, and behavior.

## Conflict of Interest Statement

The author declares that the research was conducted in the absence of any commercial or financial relationships that could be construed as a potential conflict of interest.
